# Pinocembrin alleviates ulcerative colitis in mice via regulating gut microbiota, suppressing TLR4/MD2/NF-κB pathway and promoting intestinal barrier

**DOI:** 10.1042/BSR20200986

**Published:** 2020-07-29

**Authors:** Bei Yue, Junyu Ren, Zhilun Yu, Xiaoping Luo, Yijing Ren, Jing Zhang, Sridhar Mani, Zhengtao Wang, Wei Dou

**Affiliations:** 1 Shanghai Key Laboratory of Formulated Chinese Medicines, Institute of Chinese Materia Medica, Shanghai University of Traditional Chinese Medicine (SHUTCM), Shanghai 201203, China; 2 Departments of Medicine and Genetics, Albert Einstein College of Medicine, NY 10461, U.S.A.

**Keywords:** Colitis, Gut microbiota, Intestinal barrier, Pinocembrin, TLR4/MD2/NF-κB

## Abstract

Pinocembrin, a plant-derived flavonoid, has a variety of pharmacological activities, including anti-infection, anti-cancer, anti-inflammation, cardiovascular protection, etc. However, the mechanism of pinocembrin on the anti-colitis efficacy remains elusive and needs further investigation. Here, we reported that pinocembrin eased the severity of dextran sulfate sodium (DSS)-induced colitis in mice by suppressing the abnormal activation of toll-like receptor 4 (TLR4)/nuclear factor-kappa B (NF-κB) signal pathway *in vivo*. In addition, the gut microbiota was disordered in DSS colitis mice, which was associated with a significant decrease in microbiota diversity and a great shift in bacteria profiles; however, pinocembrin treatment improved the imbalance of gut microbiota and made it similar to that in normal mice. On the other hand, *in vitro*, pinocembrin down-regulated the TLR4/NF-κB signaling cascades in lipopolysaccharide (LPS)-stimulated macrophages. At the upstream level, pinocembrin competitively inhibited the binding of LPS to myeloid differentiation protein 2 (MD2), thereby blocking the formation of receptor multimer TLR4/MD2·LPS. Furthermore, pinocembrin dose-dependently promoted the expression of tight junction proteins (ZO-1, Claudin-1, Occludin and JAM-A) in Caco-2 cells. In conclusion, our work presented evidence that pinocembrin attenuated DSS-induced colitis in mouse, at least in part, via regulating intestinal microbiota, inhibiting the over-activation of TLR4/MD2/NF-κB signaling pathway, and improving the barriers of intestine.

## Introduction

Ulcerative colitis (UC) is a chronic relasping disease of the colorectum [1]. The clinical symptoms of UC include body weight loss, diarrhea, abdominal pain, hematochezia and tenesmus [2]. UC and Crohn’s disease (CD) are the main types of inflammatory bowel disease (IBD), remaining incurable at present. The existing etiology studies, however, have not confirmed the exact pathogenesis of UC. Prevailing view suggests that UC is triggered by multiple factors, including inheritance, environment, intestinal microbiota, and innate or adaptive immunity. While UC is the result of multiple factors interaction [3,4], in this regard, the relationship between gut microbiota and UC pathogenesis is being studied extensively along with the development of sequencing technology [5]. On the other hand, a growing evidence indicates that gut microbiota dysbiosis is closely related to the occurrence of UC [6]. The imbalance of gut microbiota has been observed in UC patients in particular with a decrease in the proportion of *Firmicutes* and *Bacteroidetes*, and an increase in *Proteobacteria*, which are the three major phyla in gut [6]. Recent studies have indicated that the dysbiosis of gut microbiota could be an initiating factor in the development of UC [7]. The dysregulated microbiota damages the intestinal barrier and gives rise to increased intestinal permeability, which further activates the immune system and ultimately triggers UC [8].

The innate immune system recognizes the presence of specific bacterial antigens through pattern recognition receptors (PRRs) [9]. Toll-like receptor 4 (TLR4) is a typical member of PRRs in innate immunity, which triggers multiple signal cascades and activates the innate immune response in gastrointestinal tract by identifying pathogen-associated molecular patterns (PAMPs) and danger-associated molecular patterns (DAMPs) [10]. Compelling research has indicated that lipopolysaccharide (LPS), as a constituent of the outer membrane of Gram-negative bacteria, can bind to TLR4 and then induce the translocation of nuclear transcription factor nuclear factor-kappa B (NF-κB) into the nucleus, eventually inducing the production of cytokines and ultimately triggering inflammatory response [11]. Increased expression of TLR4 is observed in intestinal mucosa of UC patients, suggesting an important role of TLR4 in inflammatory injury [12]. Tight junction proteins (TJPs), as a continuous structure between intestinal epithelial cells, are responsible for maintaining the function of intestinal mucosal barrier. The abnormal expression of TJPs increases intestinal permeability, which makes PAMPs easier to inrush into intestinal lamina propria, eventually activating intestinal innate immunity and causing inflammation [13].

Pinocembrin ((2S)-5,7-dihydroxy-2-phenyl-2,3-dihydrochromen-4-one) is a principle bioactive flavonoid mainly isolated from propolis, honey, wild marjoram and the roots of ginger [14]. Based on pharmacological studies, pinocembrin has various biological activities in treating stroke, Alzheimer’s disease, cardiovascular diseases and atherosclerosis [15]. In addition, pinocembrin exhibits anti-inflammatory activity in macrophages and multiple inflammatory injury models [16]. A recent study revealed that pinocembrin exerts protection against DSS-induced rat colitis by increasing intestinal microflora diversity and improving barrier function [17]. However, the potency of pinocembrin on colitis mice and the underlying molecular mechanisms are not clear. In the present study, we aimed to investigate whether pinocembrin exert modulating effects on colitis mice via inhibiting the over-activation of TLR4/MD2/NF-κB signaling pathway.

## Materials and methods

### Materials

Pinocembrin (C_15_H_12_O_4_, MW: 256.25 Da, CAS: 480-39-7, HPLC purity ≥98%) was obtained from Chengdu Biopurify Phytochemicals Ltd. (Chengdu, China). RAW264.7 macrophage cell line and Caco-2 cell line were obtained from the American Type Culture Collection (Manassas, VA). Dulbecco’s modified Eagle’s medium (DMEM), Roswell Park Memorial institute (RPMI)-1640 and fetal bovine serum were purchased from Gibco BRL (Grand Island, NY). DEPC water, Lipopolysaccharide (LPS), formalin, paraformaldehyde and ethanol were from Sigma-Aldrich (St Louis, MO). Dimethyl sulphoxide (DMSO) and Tween-20 were obtained from Sangon Biotech Ltd. (Shanghai, China). Antibodies for TLR4 (ab13556), myeloid differentiation protein-2 (MD-2, ab24182) and inducible nitric oxide (iNOS, ab129372) were from Abcam (Cambridge, MA). Antibodies for ZO-1 (A11417) and Occludin (A2601) were obtained from ABclonal Technology (Wuhan, China). Antibodies against p-p65 (#SC-33039) and p-IκBa (#SC-8404) were from Santa Cruz Biotechnology (CA, U.S.A.). The other antibodies were bought from Cell Signaling Technology (Danvers, MA), as follows: p65 (#8242S), COX-2 (#12282P), TNF-α (#3707S), IκBa (#4816S). All the Quantitative polymerase chain reaction (qPCR) reagents and Reverse Transcriptase kit were from Takara Biotechnology (Shiga, Japan). Cell counting kit 8 (CCK-8) assay kit was from Dalian Meilun Biological Technology Co., Ltd. (Dalian, China). 4′,6-diamidino-2-phenylindole (DAPI) were obtained from Thermo Fisher Scientific (Waltham, MA).

### Cell lines and cell viability assay

Both RAW264.7 and Caco-2 cell lines were purchased from Shanghai Institute of Biochemistry and Cell Biology, Chinese Academy of Sciences (Shanghai, China), and all cell lines were cultured in DMEM supplemented with 10% FBS under 5% CO_2_ at 37°C. RAW264.7 cells were treated with different concentrations of pinocembrin (0–200 μM) for 24 h. CCK-8 assay was performed to measure the cytotoxic effect of pinocembrin on RAW264.7 cells. The absorption values were measured at 450 nm using a microplate reader. The cell viability was determined relative to the DMSO-treated cells.

### NO assay

RAW264.7 cells were treated with different concentrations of pinocembrin (0, 10, 25, 50, 75, 100, 150 and 200 μM) for 2 h followed by an additional treatment with LPS (1 μg/ml) for 22 h. The production of NO in the supernatant was determined by a NO assay to measure the NO secretion of each group. The absorption values were measured at 450 nm using a microplate reader as previously described [18].

### Immunofluorescence staining

RAW264.7 cells were seeded on sterility circular coverslips in a 24-well plate at a density of 4 × 10^5^ cells/well. Cells were pretreated with pinocembrin (150 μM) for 2 h followed by an additional treatment with LPS (1 μg/ml) for 22 h. Cells were washed with phosphate buffered saline (PBS) and fixed in 4% (W/V) paraformaldehyde for 10 min at room temperature. Cells were subsequently permeabilized with 0.3% (W/V) Triton X-100 for 20 min. After incubation in PBS containing 10% bovine serum albumin for 1 h at room temperature, the cells were stained with antibody against p-p65 (#3033S, Cell Signaling Technology, Danvers, MA) overnight (4°C) in the dark, then incubated with Alexa Fluor 488-conjugated secondary antibody for 1 h in the dark at room temperature. 4′,6-diamidino-2-phenylindole (DAPI) in PBS (1 μg/ml) was added to stain the nuclei. The images were captured using a fluorescence microscope (Olympus CKX41, Tokyo, Japan).

### Animals and DSS-induced colitis

Healthy C57BL/6 mice (8 weeks, 20 ± 2 g) were purchased from the Shanghai Laboratory Animal Center. All mice were maintained in specific pathogen-free facility and kept under controlled conditions at a humidity of 60–70% and stationary temperature of 23–25°C with a 12-h light/dark cycle and with access to autoclaved food and drinking water in the Laboratory Animal Center of the Shanghai University of Traditional Chinese Medicine. All animal experiments were conducted with the principles of the declaration and recommendations of the Animal Experimentation Ethics Committee at Shanghai University of Traditional Chinese Medicine (Animal license No: SZY201603004).

Acute experimental colitis was induced in mice by administering 3.5% DSS (MW: 36,000-50,000 Da, MP Biochemicals, Irvine, CA) in the drinking water as described previously [19]. C57BL/6 mice were randomly divided into the following groups (*n*=6): vehicle group, pinocembrin group (100 mg/kg of body weight), DSS group, and DSS + pinocembrin group (25, 50 and 100 mg/kg of body weight). The DSS group received 3.5% DSS in drinking water for 7 consecutive days. Pinocembrin was dissolved in 0.5% methylcellulose and administered by daily oral gavage 2 days prior to DSS treatment and continued to the end of the DSS treatment.

### Clinical and histological assessment of colitis

Body weight and bloody diarrhea score were evaluated and recorded daily. Bloody stool score was evaluated according to the following standards: normal-colored stool (score 0), brown stool (score 1), reddish stool (score 2), bloody stool (score 3) [20]. All groups were anesthetized under pentobarbital sodium anesthesia and euthanized by cervical dislocation. After euthanasia, the entire colon was removed and the colon length was measured. Then distal colon was fixed in 10% paraformaldehyde and embedded in paraffin. Tissue sections were stained with hematoxylin and eosin (H&E) for histological evaluation. Histological injury was assessed by a combined score of inflammatory cell infiltration (score 0–3) and epithelial damage (score 0–3) using a double-blind method as described previously [21].

### Immunoblotting and RNA analysis

Colon segments or cultured cells were homogenized in PBS or lysed in lysis buffer containing protease and phosphatase inhibitor cocktail tablets (Roche Diagnostics GmbH, Mannheim, GER). The supernatant was collected after centrifugation (4°C, 12000 ***g***, 15 min). Proteins (30 μg) was separated by 10% SDS-PAGE and transferred onto a PVDF membrane. The membrane was blocked in 5% (w/v) skim milk for 2 h at room temperature and immunoblotted with primary antibody. Then blots were washed and incubated with HRP-coupled secondary antibody at room temperature. Finally, the blots were observed by enhanced chemiluminescence (ECL) detection reagents. Protein expressions were analyzed by a GS-700 imaging densitometer (Bio-Rad, CA). β-actin (Santa Cruz) was used as an internal control.

Total RNA was extracted from cultured cells and colon samples using a TRIzol reagent following the instructions. The complementary DNA (cDNA) was reversely transcribed from 3 μg of total RNA using the SuperScript III Reverse Transcriptase kit. Quantitative polymerase chain reaction (qPCR) was carried out using SYBR Premix ExTaq Mix and quantitatively measured with an ABI Prism 7900HT Sequence Detection System (Life Technologies, Carlsbad, CA). The following thermal cycler parameters were used: 1 cycle of 95°C for 30 s and 40 cycles of denaturation (95°C, 5 s) and combined annealing/extension (60°C, 30 s). The relative expression of mRNA was normalized as the ratio of optimal density relative to β-actin. The PCR primers are listed in Table 1.

**Table 1:** The list of primers

Gene	Primer sequence
m TLR4	F: GTCCTACACCACACCAAACT
	R: ATCTCTGCCTATCCGTCTC
m MyD88	F: ACAACAA0CTCCATCCTCCT
	R: GGTATTTCATCTCTCTGCTCTG
m TNF-α	F: CTCTTCTCATTCCTGCTTGT
	R: GTGGTTTGTGAGTGTGAGG
m iNOS	F: ATTGTGGCTGTGGAGAAG
	R: AAGATGAAGGAAAAGAAGGTG
m COX-2	F: GCCTTCCCTACTTCACAA
	R: ACAACTCTTTTCTCATTTCCAC
m IFN-γ	F:AGCAACAACATAAGCGTCAT
	R:CCTCAAACTTGGCAATACTC
m IL-6	F:GCCTTCCCTACTTCACAA
	R:ACAACTCTTTTCTCATTTCCAC
m IL-15	F:ATGAAAATTTTGAAACCATATATGA
	R:CAAGACGTGTTGATGAACATTTGGA
m β-actin	F:GGGAAATCGTGCGTGAC
	R: AGGCTGGAAAAGAGCCT
h Claudin-1	F: TGGAGACCTGGATTTGAGTC
	R: AACCACCGCTTACAGATGAA
h Occludin	F: CATTGCCATCTTTGCCTGTG
	R: AGCCATAACCATAGCCATAGC
h JAM-A	F: GTGAAGTTGTCCTGTGCCTACTC
	R: ACCAGTTGGCAAGAAGGTCACC
h β-Actin	F:GACATCCGCAAAGACCTG
	R:GGAAGGTGGACAGCGAG

### Microbiota sequence analysis

Genomic DNA was extracted from the fecal samples using the E.Z.N.A.® soil DNA Kit (Omega Bio-tek, Norcross, GA, U.S.A.) according to the manufacturer’s protocol. The concentration and quality of DNA were checked using NanoDrop 2000 UV-vis spectrophotometer (Thermo Scientific, Wilmington, DE, U.S.A.). Then, the bacteria’s 16S rRNA gene in V3-V4 hypervariable regions were amplified with primers 338F (5′-ACTCCTACGGGAGGCAGCAG-3′) and 806R (5′-GGACTACHVGGGTWTCTAAT-3′) by thermocycler PCR system (GeneAmp 9700 ABI, Carlsbad, CA, U.S.A.). The amplification procedures were conducted at 95°C for 3 min in the first place, followed by 27 cycles (denaturation at 95°C for 30 s, annealing at 55°C for 30 s, and elongation at 72C for 45 s) and a final extension (72°C, 10 min). Illumina MiSeq sequencing was further processed using the high throughput Illumina MiSeq platform (Illumina, San Diego, CA, U.S.A.) according to the standard protocols (Majorbio, Shanghai, China). The sequencing raw fastq files were quality-filtered by Trimmomatic and merged by FLASH with the criteria as previously reported [22]. UPARSE [23] with a novel ‘greedy’ algorithm was carried out to perform the operational taxonomic units (OTUs) clustering with 97% similarity cutoff. The taxonomy of each 16S rRNA gene sequence was analyzed by RDP Classifier algorithm against the Silva (SSU123) 16S rRNA database using a 70% confidence threshold.

### Molecular docking

The docking experiment was carried out with AutoDock 4.2.6 program to investigate the combining ability between pinocembrin and MD2 protein as described previously [18,24]. The available 3D structures of MD2 protein (PDB code: 2E56 [25], resolution: 2.0 Å) were obtained from the Protein Data Bank. The initial structure was prepared using AutoDockTools 1.5.6 [26], preserving the original charge of the protein and generating a pdbqt file for docking. The 2D structure of the Pinocembrin was downloaded from the PubChem database. MOPAC program was then used to optimize the structure and calculate the PM3 atomic charge [27,28]. The structure of Pinocembrin was also prepared by AutoDock Tools 1.5.6, and the corresponding pdbqt file was generated for docking. The hydrophobic region of MD2 was chosen as the binding pocket for docking. The coordinates of grid box center were (2.45, 24.00, 13.22), and the number of grid points in the XYZ of grid box was set to 80 × 60 × 60. The grid spacing was 0.375 Å, and the number of GA run was set to 100. The rest parameters were set to default.

### Immunoprecipitation assay

The immunoprecipitation assay was carried out using a Dynabeads™ Protein A Immunoprecipitation Kit (Lot. 00686627, invitrogen, Thermo Fisher Scientific, Lithuania) according to the operation manual. RAW264.7 cells were lysed in lysis buffer containing protease and phosphatase inhibitor cocktail tablets. A sufficient amount of TLR4 antibody (2 μg) was added to 500 μg cellular protein, and then protein samples were incubated and gently rotated for 2 h at 4°C. Ultimately, the immune complexes were precipitated with protein A beads (20 μl, 4°C, overnight), and the precipitates were washed with IP buffer (supplied in kit) for three times. The protein samples were then boiled in sample buffer, followed by immunoblotting to analysis the expression of TLR4 and MD2.

### Immunohistochemistry

The paraffin-embedded colonic tissue slides were incubated with antibodies against mouse TLR4 (diluted 1:50) overnight at 4°C. After further washing, the slides were incubated with Envision/HRP (diluted 1:250) at 37°C for 30 min. Finally, immune complexes were visualized by incubating with diaminobenzidine for 10 min and counterstained with hematoxylin.

### NF-κB luciferase reporter assay

The pGL4.32 [luc2P/NF-κB-RE/Hygro] luciferase reporter vector (Promega, Madison, WI) was electroporated into RAW264.7 cells by using a Lonza Nucleofector II instrument (Amaxa Biosystems, Germany) as described previously [18]. Cells were incubated with pinocembrin (0–150 µM) for 2 h and followed by an additional treatment with LPS (1 μg/ml) for 22 h. Cells were washed with PBS and then lysed in 1× passive lysis buffer (100 µl). The luciferase activity from cell lysates was detected using a luciferase assay system (Promega, Madison, WI). Results were expressed as fold induction of control cells.

### Statistics

Statistical analysis was conducted to compare among multiple groups using one-way analysis of variance (ANOVA) by GraphPad Prism 7 software (GraphPad Software, La Jolla, CA, U.S.A.). Data was calculated as the Mean ± SD. *P*-values < 0.05 (two-sided) were considered significant (*P<0.05, **P<0.01, ***P<0.001). All the 16S rRNA sequencing data were analyzed on the online Majorbio I-Sanger Cloud Platform (www.i-sanger.com).

## Result

### *In vivo* study

#### Pinocembrin ameliorated clinical symptoms of DSS-induced colitis

**Figure 1:**
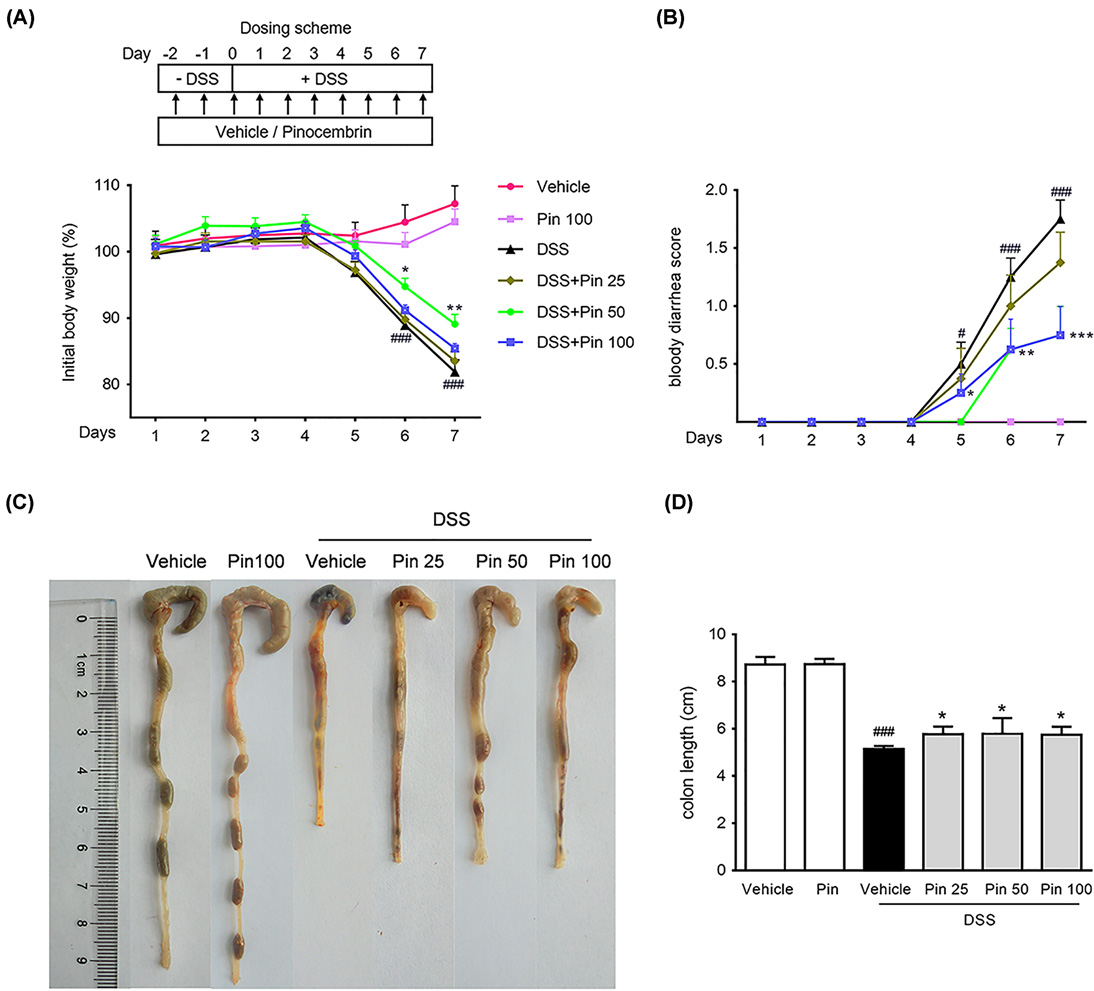
Treatment with pinocembrin ameliorated colitis symptoms in mice (**A**) Body weight was recorded following DSS induction of colitis. Data were plotted as a percentage of basal body weight. (**B**) The score of bloody diarrhea. Data were plotted as the scores that had bloody diarrhea at different time points of DSS treatment. Macroscopic observation (**C**) and assessment of colon shortening (**D**) after DSS treatment. Data were expressed as the mean ± SD (*n*=6 mice per group). “Pin” is equal to pinocembrin, and that the doses are in mg/kg, and that the doses are in mg/kg. *P<0.05, **P<0.05, ***P<0.001 vs. DSS group; ^#^*P*<0.05, ^##^*P*<0.01, ^###^*P*<0.001 vs. Vehicle group.

**Figure 2:**
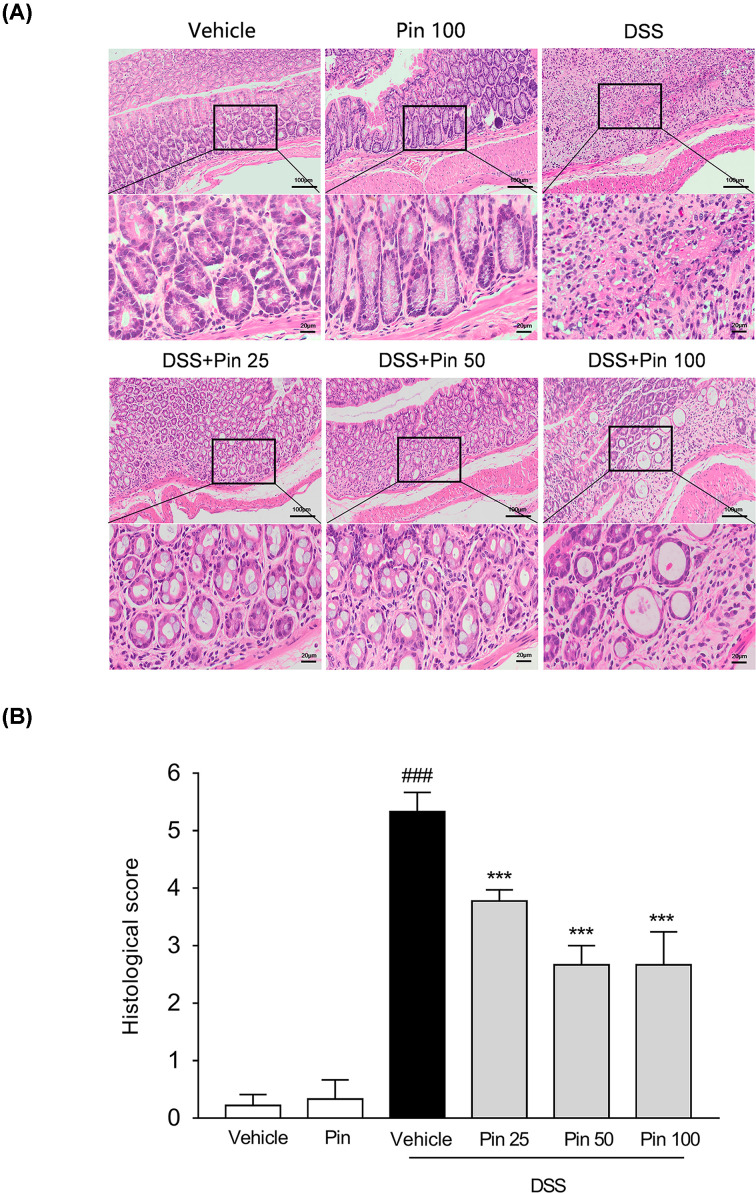
Pinocembrin ameliorated inflammatory infiltration and histopathological injury in DSS-treated mice Representative H&E-stained colon sections (**A**) and histological score (**B**). Scale bar corresponds to 100 or 25 μm. Data were expressed as the mean ± SD (*n*=6 mice per group). ***P<0.001 vs. DSS group; ^###^*P*<0.001 vs. Vehicle group.

The typical features in DSS-induced mouse model of UC are continuous body weight loss, diarrhea and blood in stool [29]. As expected, the DSS alone treatment group showed a dramatic body weight reduction and diarrhea, but mice treated by pinocembrin (50 or 100 mg/kg) had significantly slighter colitis symptoms (Figure 1A,B). Furthermore, colon shortening is an indirect marker indicating the intensity of inflammation in colon [30]. As anticipated, the DSS-induced colon shortening was remarkable, but it was significantly mitigated by pinocembrin (25–100 mg/kg) treatment (Figure 1C,D). The pathological histology analysis showed that DSS caused severe intestinal tissue damage, including severe epithelial injury, architecture loss, muscle thickening and neutrophil infiltration. Pinocembrin (25–100 mg/kg) treatment mitigated mucosal architecture loss, ulcerations and cellular infiltration (Figure 2A,B). In particular, 50 mg/kg of pinocembrin was the optimal dose according to the mice study. Hence, 50 mg/kg treatment group was used in the subsequent analyses.

#### Pinocembrin regulated gut microbiota composition

**Figure 3:**
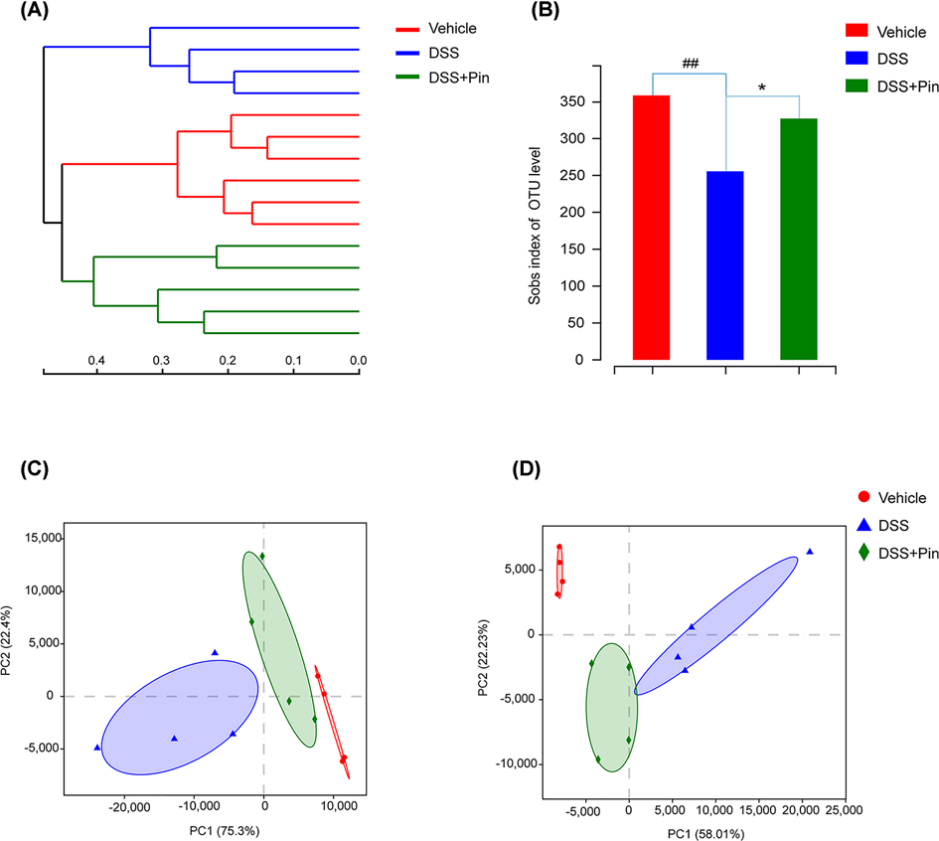
Pinocembrin regulated the composition of gut microbiota in DSS-treated mice (**A**) Hierarchical clustering tree. Classification level: OUT; Distance Algorithms: Bray-Curtis Dissimilarity; Sample Hierarchical Clustering: Complete-Linkage Clustering. (**B**) Index-group Difference Test of Sobs Value in Sample Hierarchical Cluster Tree α-Diversity. Student’s *t*-test. (**C**) Principal Component Analysis (PCA) graphical result in Phylum level. (**D**) Principal Component Analysis graphical result in Genus level. Data = mean ± SD (*n*=4 mice per group). *P<0.05 vs. DSS group; ^##^*P*<0.01 vs. Vehicle group.

**Figure 4:**
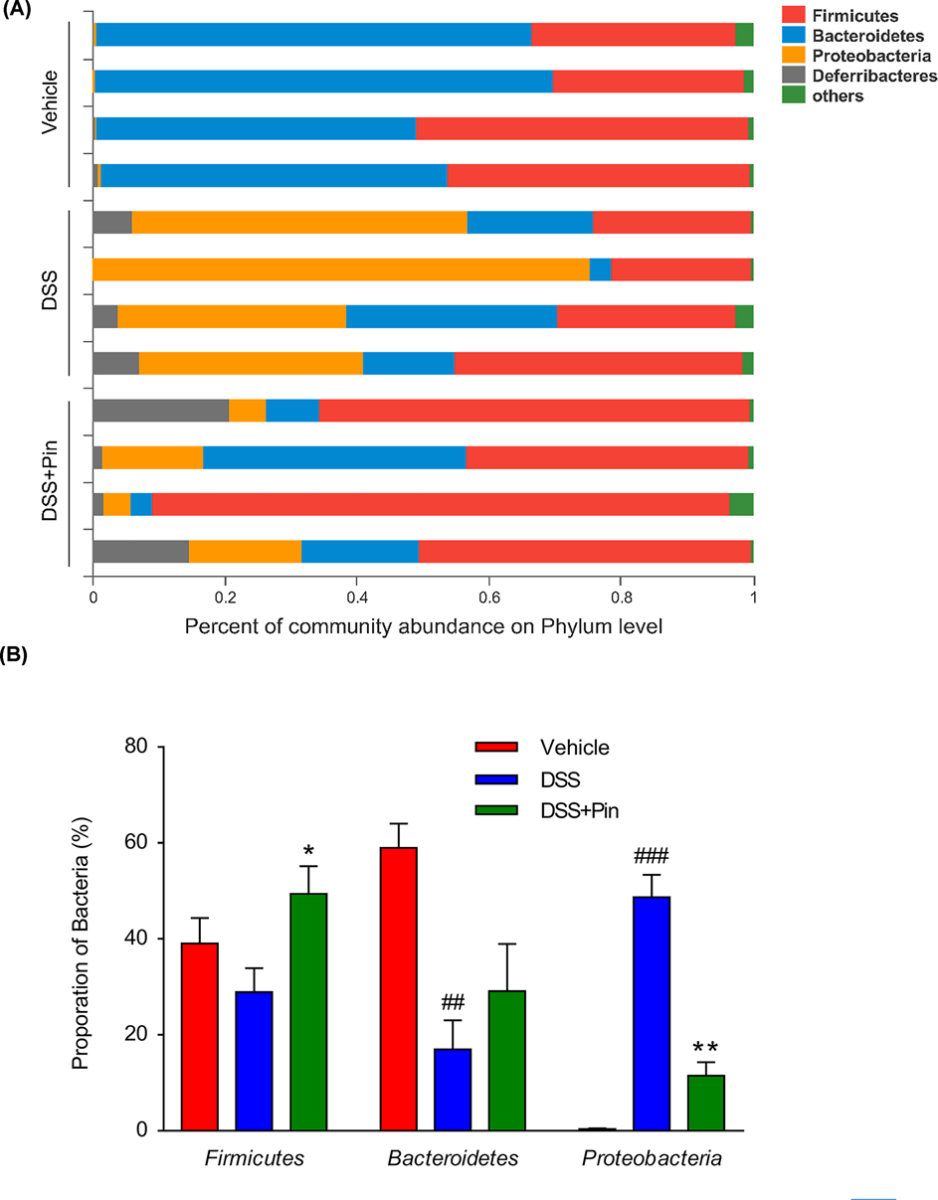
Pinocembrin regulated the gut microbiota composition and abundance in Phylum level (**A**) The proportion of dominant Phylum communities in each group of samples. Phylum communities less than 10% was merged into others. (**B**) Distribution of three dominant Phylum (*Firmicutes, Bacteroidetes* and *Protebacteria*) in each groups. Data were exhibited as the mean ± SD (*n*=4 mice per group). *P<0.05, **P<0.01 vs. DSS group; ^##^*P*<0.01, ^###^*P*<0.001 vs. Vehicle group.

**Figure 5:**
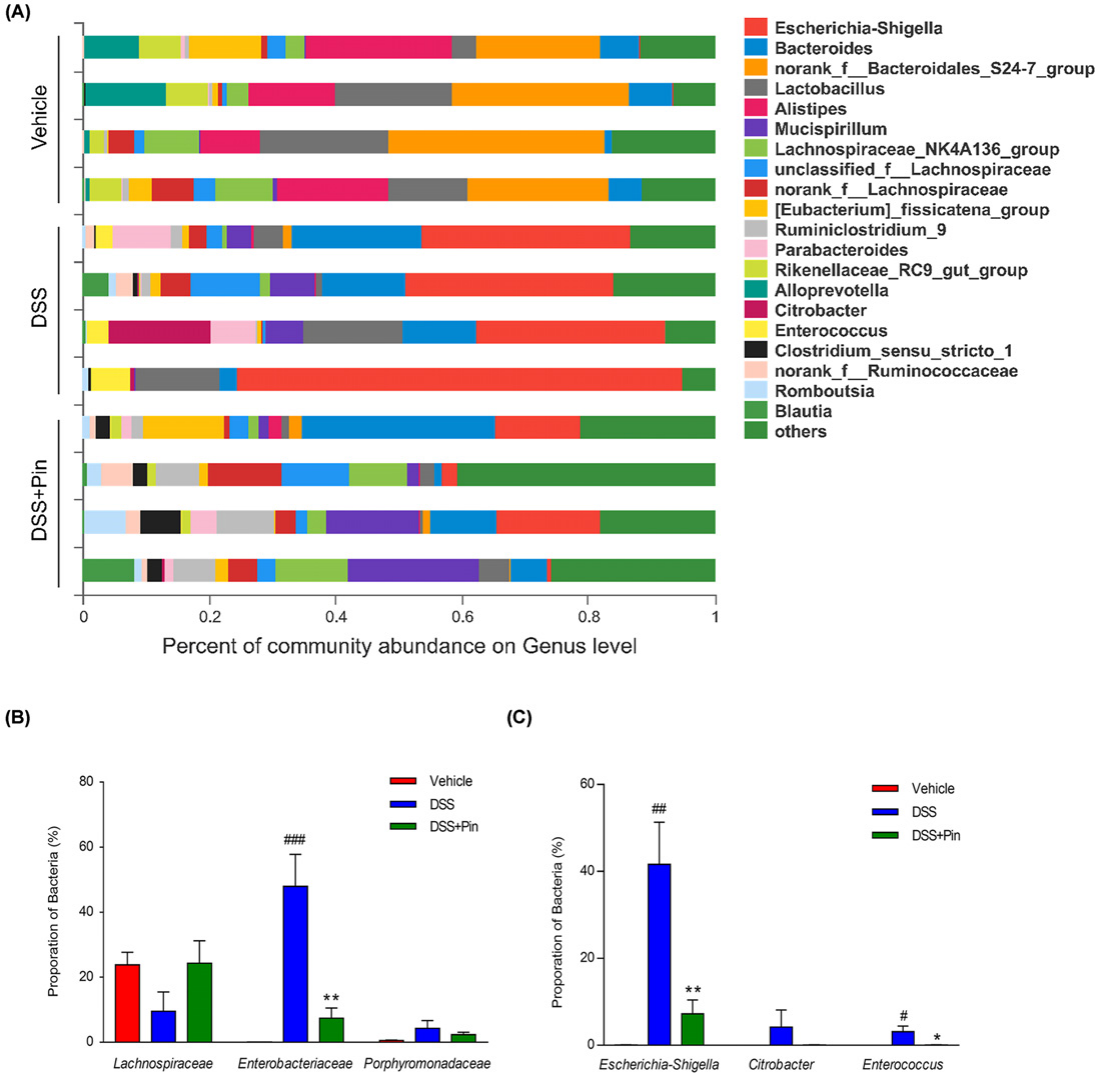
Pinocembrin regulated the composition and abundance of gut microbiota in Genus level (**A**) The proportion of dominant Genus communities in each group of samples. Genus communities less than 5% was merged into others. (**B**) Distribution of three dominant Family (*Lachnospiraceae, Enterobacteriaceae* and *Porphyromonadaceae*) in each groups. (**C**) Distribution of three dominant Genus (*Escherichia-Shigella, Citrobacter* and *Enterococcus*) in each groups. Data were exhibited as the mean ± SD (*n*=4 mice per group). *P<0.05, **P<0.01 vs. DSS group; ^#^*P*<0.05, ^##^*P*<0.01, ^###^*P*<0.001 vs. Vehicle group.

16S rRNA sequencing results were analyzed to evaluate the impact of pinocembrin on the altered gut microbiota composition induced by DSS. The hierarchical clustering tree on OUT level and principal component analysis (PCA) revealed a clearly separate cluster for each group (Figure 3A,C,D). The Shannon index was used to indicate the microbial diversity. As it was shown in Figure 3B, DSS-treated mice revealed a significant decrease in microbial diversity, whereas pinocembrin treatment group alleviated these changes. The major intestinal bacteria in phylum level, exhibited in the diagram, including *Firmicutes, Bacteroidetes, Proteobacteria* and *Deferribacteres.* Moreover, DSS treatment decreased the relative abundance of *Firmicutes* and *Bacteroidetes* and enriched the abundance of *Proteobacteria* compared with normal control group (Figure 4A). However, pinocembrin treatment mitigated the DSS-induced changes in phylum level (Figure 4B). At genus level of taxonomic criteria, the DSS model group exhibited a proportional exhaustion for *Lachnospiraceae*, which was improved by pinocembrin treatment (Figure 5A). In addition, there was a significant growth in pathogenic bacteria in DSS model mice, such as the enrichment of *Enterobacteriaceae* and *Porphyromonadaceae* at the family level, which was also suppressed by pinocembrin (Figure 5A,B). Furthermore, some pathogenic bacteria in DSS model group were increased significantly, such as *Escherichia-Shigella* and *Enterococcus*, whereas the abundance of *Escherichia-Shigella* and *Enterococcus* was decreased by pinocembrin treatment (Figure 5A,C). Moreover, there are also slight changes in *Porphyromonadaceae* and *Citrobacter* between the DSS model group and the DSS group with obacunone treatment, athough no significant differences is observed (Figure 5B,C). Collectively, the data indicated that gut microbiota homeostasis could be disturbed by DSS administration, while pinocembrin treatment could reverse DSS-induced gut microbiota imbalance.

#### Pinocembrin suppressed TLR4/NF-κB signaling cascades in DSS colitis mice

**Figure 6:**
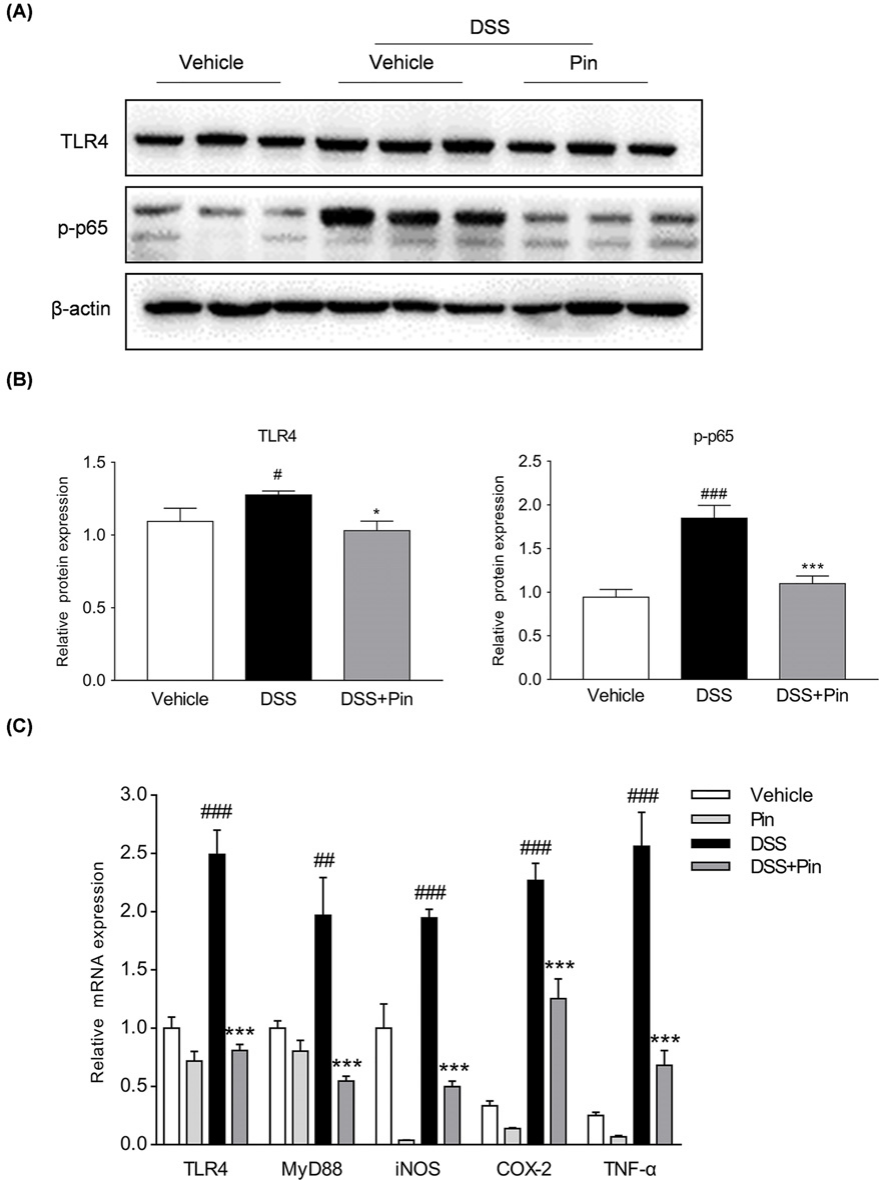
Pinocembrin inhibited TLR4/NF-κB signaling *in vivo* (**A**) Colon segments from mice were excised and homogenized, and the total protein was subjected to immunoblotting with antibodies against TLR4, p-p65 (1:1000 dilution) and β-actin (1:2000). One representative blot was shown. (**B**) All the expressions were normalized to β-actin. Quantification of the protein expression was performed by densitometric analysis of the blots. (**C**) mRNA expression of TLR4, MyD88, iNOS, COX-2 and TNF-α in colonic tissue were determined by qRT-PCR. All the expressions were normalized to β-actin. Data were exhibited as the mean ± SD (*n*=3). *P<0.05, ***P<0.001 vs. DSS group; ^#^*P*<0.05, ^##^*P*<0.01, ^###^*P*<0.001 vs. Vehicle group.

**Figure 7:**
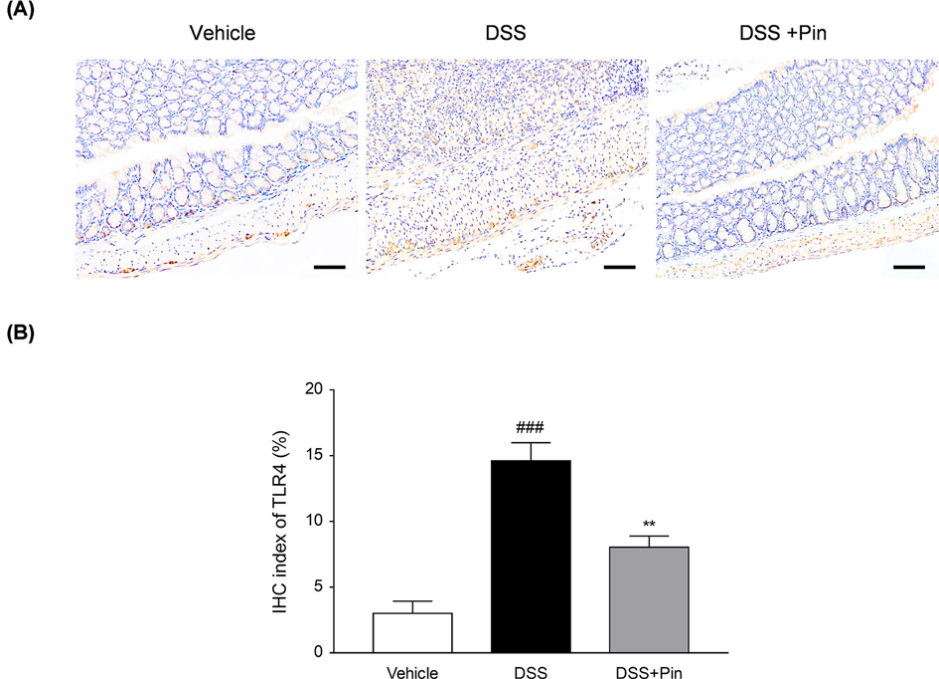
Pinocembrin restrained TLR4 expression *in vivo* (**A**) Immunochemical staining of TLR4 in colonic tissue sections. Scale bar, 100 μm. (**B**) Quantitative determination of immunochemical (IHC) index of TLR4 positive signals. Data were exhibited as the mean ± SD (*n*=3). **P<0.01 vs. DSS group; ^###^*P*<0.001 vs. Vehicle group.

Excessive activation of TLR4/NF-κB signaling cascades plays a vital role in UC mice [2]. To investigate the underlying relevance of pinocembrin on TLR4/NF-κB signaling during DSS-induced colitis, we evaluated the influence of pinocembrin on the activation of TLR4 and the phosphorylation of p65 by immunoblotting. A significant increase in TLR4 protein and the phosphorylation of p65 (p-p65) level was observed in the colon of DSS-induced mice (Figure 6A,B). However, administration of pinocembrin greatly reduced the levels of TLR4 and p-p65 in the inflamed colon. In the meanwhile, we observed that mRNA expression of TLR4, Myd88, iNOS, COX-2 and TNF-α in colonic tissue was significantly increased following DSS treatment (Figure 6C). Nevertheless, pinocembrin significantly inhibited the increased levels of TLR4, Myd88, iNOS, COX-2 and TNF-α in inflamed colon. On the other hand, immunohistochemistry staining showed that DSS treatment led to a pronounced increase in TLR4 positive signals in the colonic tissue. However, treatment with pinocembrin markedly reduced the TLR4 expression in the colon (Figure 7A,B).

### *In vitro* study

#### Pinocembrin attenuated pro-inflammatory mediators in macrophages

**Figure 8:**
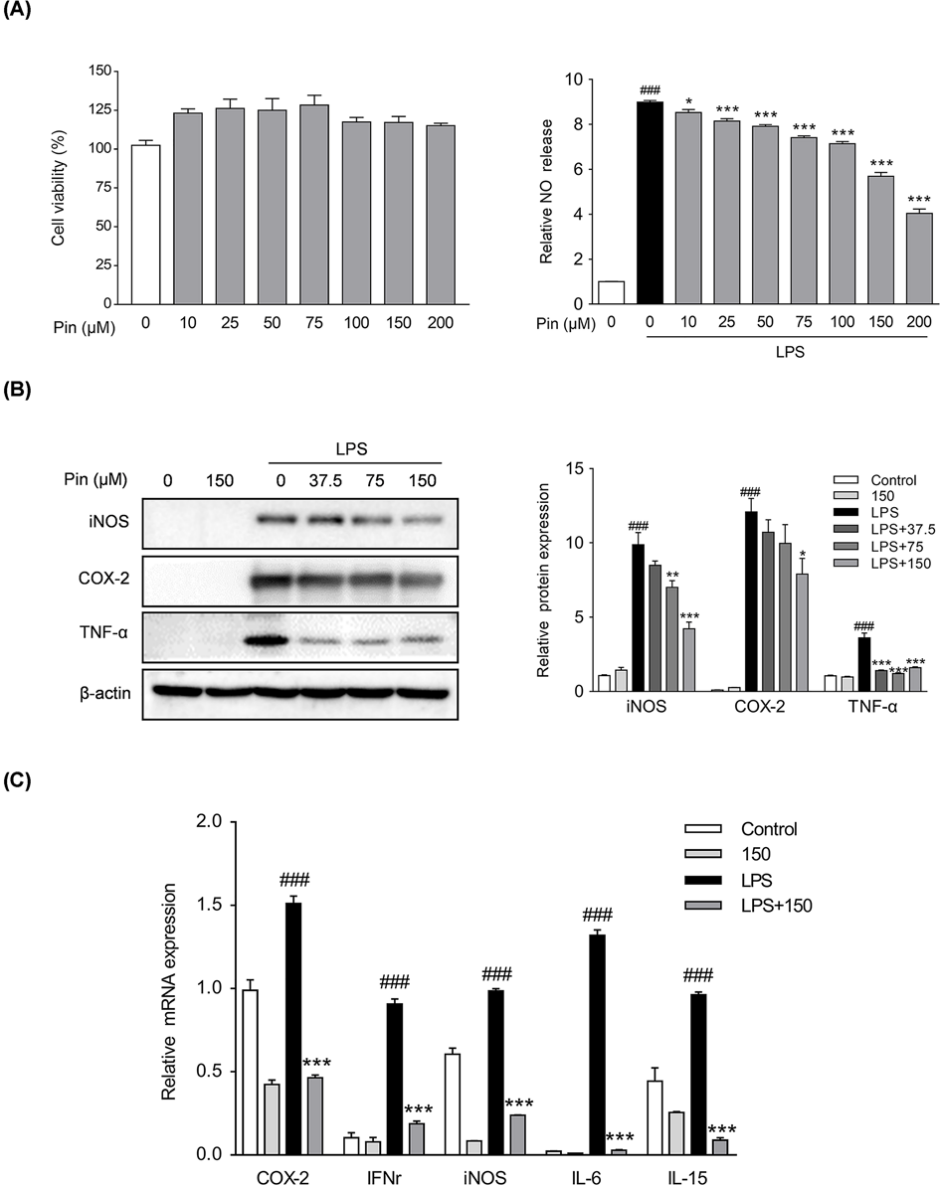
Pinocembrin inhibited the expression of pro-inflammatory mediators *in vitro* (**A**) Effect of pinocembrin on cell viability and the secretion of NO in LPS-induced RAW264.7. Cells were treated with pinocembrin (0–200 μM) for 24 h, and then cell viability was measured. Also, the production of NO was determined. (**B**) RAW264.7 cells were treated as described in ‘Materials and Methods’ section. Protein expression levels of iNOS, COX-2 and TNF-α were determined by immunoblotting analysis. Expression was normalized to β-actin. (**C**) Effect of pinocembrin on the expression of pro-inflammatory mediators mRNA in LPS-induced RAW264.7 cells. All the expressions were normalized to β-actin. Data were exhibited as the mean ± SD (*n*=3). *P<0.05, **P<0.01, ***P<0.001 vs. LPS group; ^###^*P*<0.001 vs. Vehicle group.

Macrophages are the major source of pro-inflammatory cytokines when inflammation occurs in the intestine [31]. Therefore, we used RAW264.7 mouse macrophages to evaluate the anti-inflammatory effects of pinocembrin *in vitro*. A cytotoxicity test suggested that pinocembrin had no obvious effects on the cell viability at dosage up to 200 μM (Figure 8A). Moreover, the LPS-stimulated production of NO in RAW264.7 cells was restrained by pinocembrin treatment in a concentration dependent manner at dosage up to 200 μM (Figure 8A). Thus, the concentrations of 37.5, 75 and 150 μM were selected for use in our further investigation. Consistent with suppression of NO production, a significant increase in the protein level of iNOS was observed in RAW264.7 cells exposed to LPS, and treatment with pinocembrin reduced the iNOS level in a dose-dependent manner (Figure 8B). Moreover, the excessive secretion of pro-inflammatory mediators are critical inflammatory factors that induce multiple inflammatory disorders [32]. We therefore evaluated the effects of pinocembrin on pro-inflammatory mediators expression. As displayed in Figure 8C, the expression levels of TNF-α, COX-2, iNOS, IFN-γ, IL-6 and IL-15 were markedly upregulated in LPS-stimulated macrophages. However, treatment with pinocembrin reduced the levels of pro-inflammatory mediators in LPS-stimulated macrophages.

#### Pinocembrin attenuated the activation of TLR4/NF-κB signaling in macrophages

**Figure 9:**
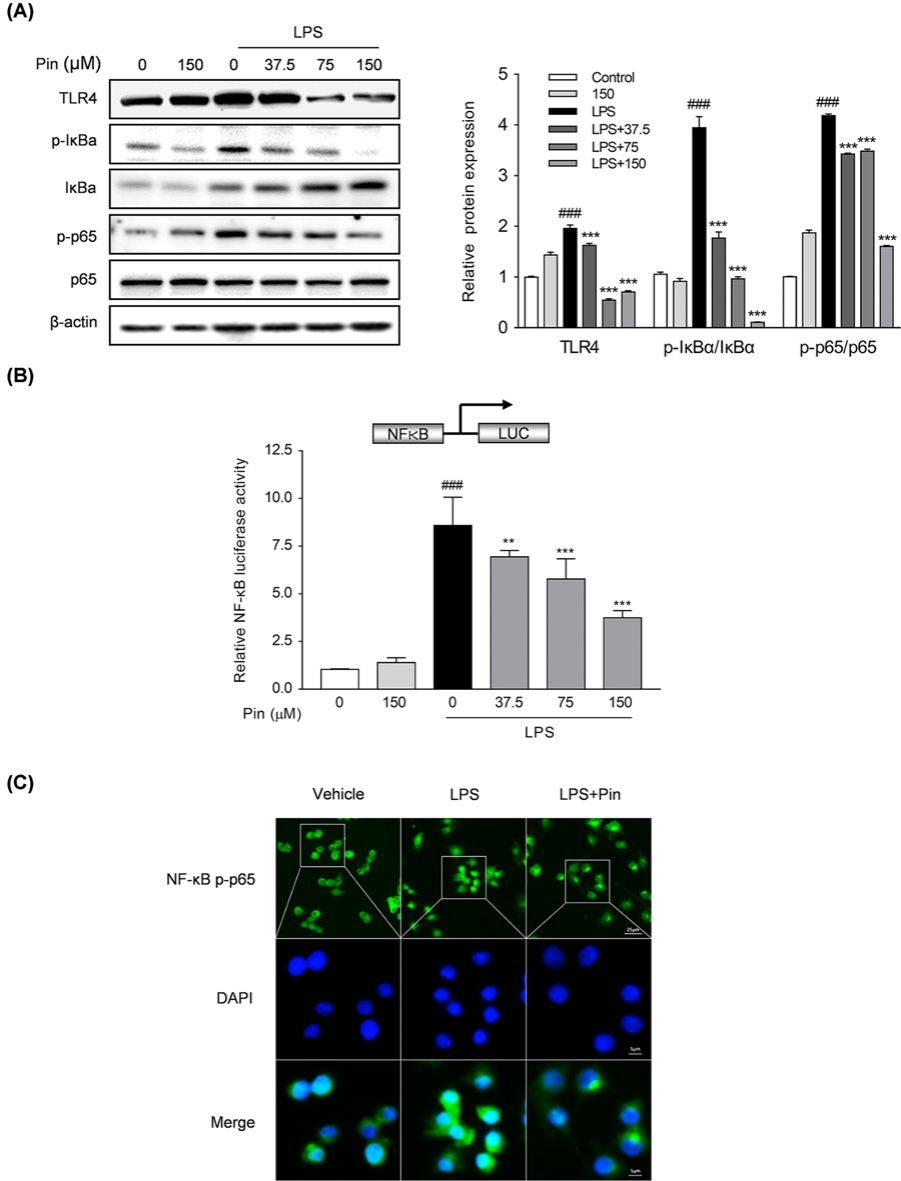
Pinocembrin inhibited TLR4/NF-κB signaling *in vitro* RAW264.7 cells were exposed to pinocembrin (150 μM) for 2 h and then treated with LPS (1 μg/ml) for an additional 22 h. (**A**) Effect of pinocembrin on TLR4 pathway-related proteins expression in LPS-treated RAW264.7 cells was determined by immunoblotting analysis. (**B**) RAW264.7 cells were transfected with pGL4.32[luc2P/NF-κB-RE/Hygro] luciferase reporter vector and cell extracts were assayed for luciferase activity as described in the ‘Materials and Methods’ section. (**C**) Immunofluorescence staining was conducted to detect the NF-κB p-p65 nuclear translocation. Scale bar corresponds to 25 or 5 μm. Data were exhibited as the mean ± SD (*n*=3). **P<0.01, ***P<0.001 vs. LPS group; ^###^*P*<0.001 vs. Vehicle group.

To provide further insight into the anti-inflammatory mechanisms of pinocembrin, the activation of TLR4/NF-κB signaling molecules in RAW264.7 cells was evaluated by immunoblotting. As shown in Figure 9A, the expression level of TLR4, phosphorylation of p-65 and phosphorylation/degradation of IκBα were obviously augmented after LPS stimulation, which were down-regulated by pinocembrin treatment in a concentration-dependent manner. To prove the direct involvement of TLR4/NF-κB signaling, we treated RAW264.7 cells with TLR4 ligand LPS and measured NF-κB transcriptional activity. As shown in Figure 9B, pinocembrin dose-dependently decreased the LPS-induced NF-κB-luciferase activity. Furthermore, the LPS-induced nuclear translocation of p-p65 was also repressed by pinocembrin treatment (Figure 9C).

#### Pinocembrin blocked the formation of receptor multimer TLR4/MD2·LPS

**Figure 10:**
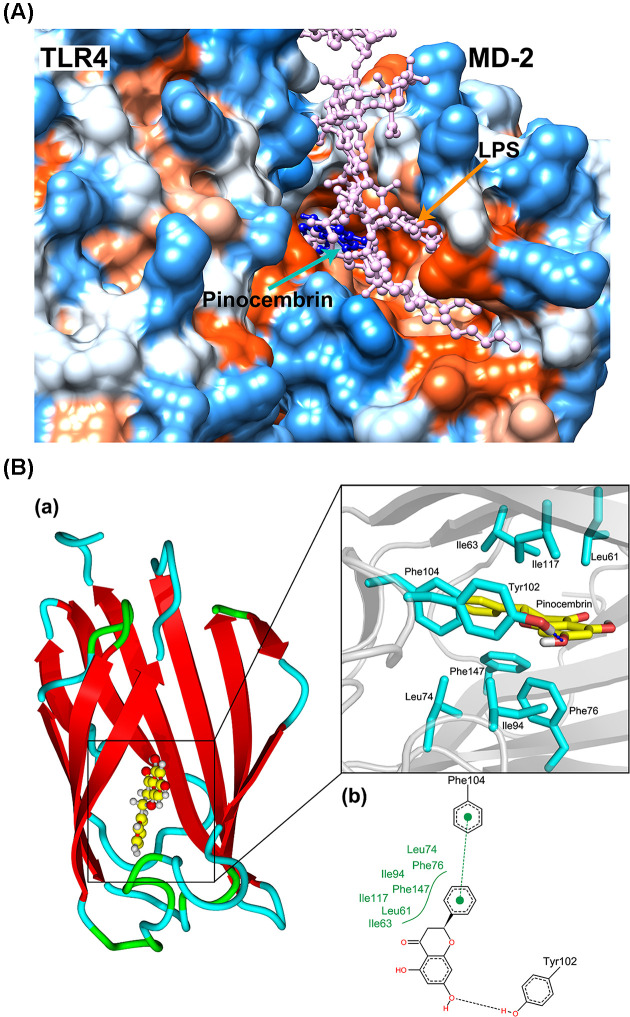
Pinocembrin blocked the binding of LPS to MD2 protein by competitive occupancy with LPS (**A**) Hydrophobic matching map of Pinocembrin (blue), LPS (pink) and TLR4/MD2 complex. (blue and orange areas on the protein surface indicate hydrophilic and hydrophobic regions, respectively). (**B**) The binding mode of Pinocembrin and MD2. (a: yellow stick represents Pinocembrin, cyan stick indicates the residues involved in interactions, hydrogen bond is shown in blue dash line; b: π–π interaction is shown in green sphere and dash line, the black dotted line indicates hydrogen bond, and the green curve indicates hydrophobic interactions)

Myeloid differentiation protein 2 (MD2), an indispensable accessory protein, directly recognizes the lipid A domain of LPS, inducing the formation of TLR4/MD2 protein complex. The formation of TLR4/MD2 association eventually leads to the recruitment of signaling adapter MyD88 and subsequent activation of downstream immune responses [33]. Therefore, suppressing TLR4 signaling by targeting MD2 is indicated to be a potential way to inhibit inflammatory response [34]. In the present study, molecular docking analysis was performed to investigate the potential combination mechanism of pinocembrin binding to MD2. As shown in Figure 10A, pinocembrin was fitted into the hydrophobic pocket of MD2, and apparently overlapped with the hydrophobic lipid tail of LPS. The 3D and 2D interaction diagrams of pinocembrin-MD2 complex were shown in Figure 10B, from which, pinocembrin was indicated to mainly bind to the hydrophobic pocket of MD2 formed by Leu61, Ile63, Leu74, Phe76, Ile94, Tyr102, Phe104, Ile117 and Phe147, and formed a hydrogen bond and π–π interaction with Tyr102 and Phe104, respectively. These interactions further enhanced the affinity between pinocembrin and MD2 protein. Therefore, it was speculated that the binding of pinocembrin to MD2 may competitively inhibit the recognition of LPS by TLR4.

**Figure 11:**
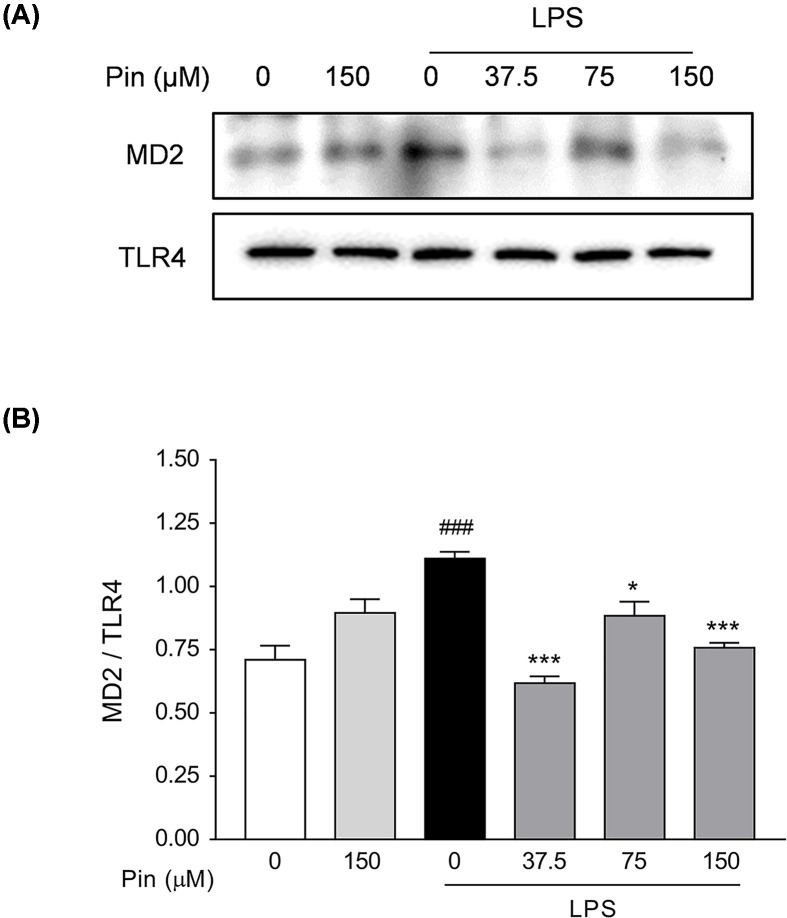
Pinocembrin blocked LPS-induced TLR4/MD2 association (**A** and** B**) Co-immunoprecipitation assay was carried out to investigate the effect of pinocembrin on the formation of TLR4/MD2 complex in RAW264.7 cells. RAW264.7 cells were pretreated with pinocembrin (0–150 μM) for 2 h and then stimulated with LPS (1 μg/ml) for 22 h. Ultimately, cell lysates were immunocoprecipitated with anti-TLR4 antibody and then subjected to immunoblotting analysis of MD2 and TLR4. The immunocoprecipitative immunoblots were analyzed by densitometric analysis of the MD2/TLR4 ratio. Data were expressed as the mean ± SD (*n*=3). *P<0.05, ***P<0.001 vs. LPS group; ^###^*P*<0.001 vs. Vehicle group.

To further elucidate the influence of pinocembrin on the formation of LPS-induced TLR4/MD2 complex, co-immunoprecipitation experiment was carried out. As illustrated in Figure 11A,B, LPS treatment prominently enhanced the co-precipitation of TLR4/MD2 complex, while pinocembrin treatment markedly inhibited the formation of LPS-induced TLR4/MD2 association.

These data indicated that pinocembrin bound directly to MD2 protein, blocked the formation of multimer TLR4/MD2·LPS, and subsequently interrupted the downstream inflammatory signaling.

#### Pinocembrin up-regulated tight junction proteins in intestinal epithelial cells

**Figure 12:**
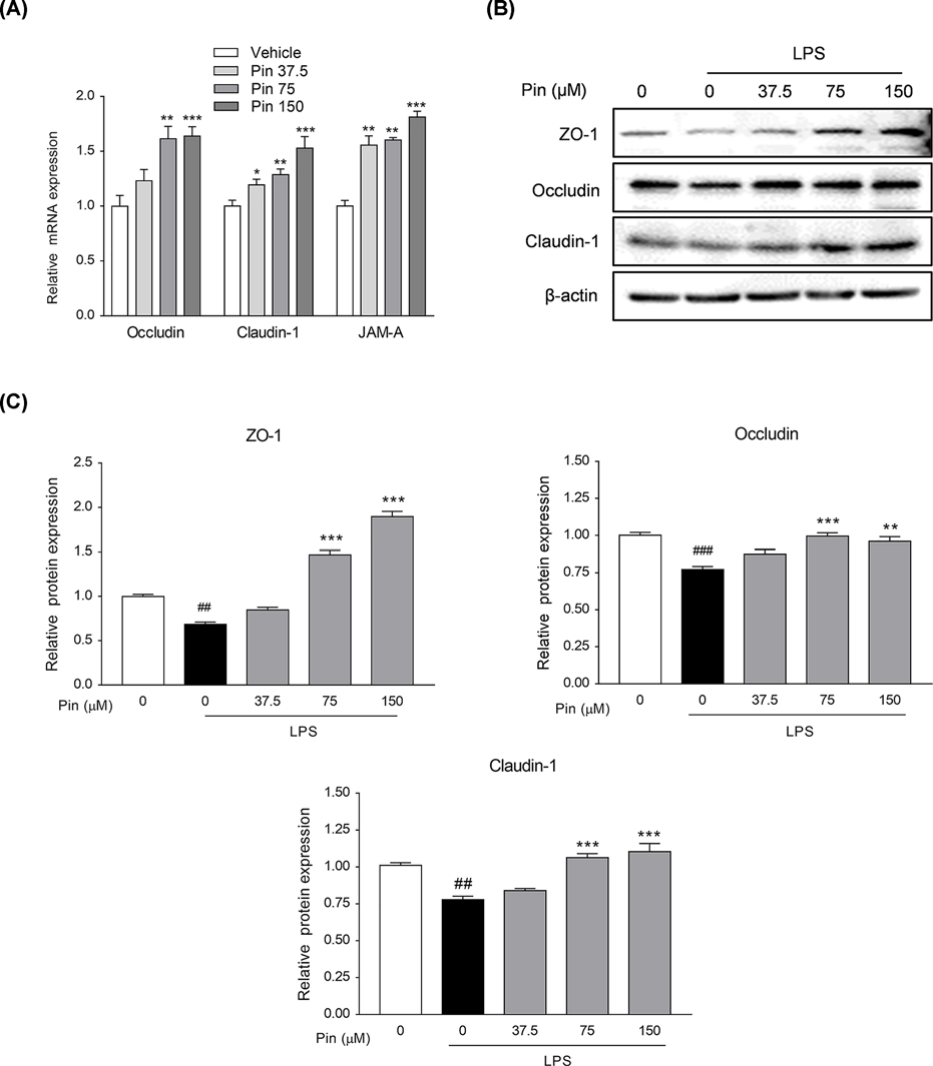
Pinocembrin increased the expression of tight junction proteins *in vitro* (**A**) Caco-2 cells were treated with different concentration of pinocembrin (0–150 μM) for 24 h, and then the mRNA expression of Occludin, Claudin-1 and JAM-A in Caco-2 cells were determined by qRT-PCR. (**B** and** C**) Caco-2 cells were treated with pinocembrin for 2 h prior to LPS (1μg/ml) treatment for an additional 22 h. Protein expression of ZO-1, Occludin and Claudin-1 in Caco-2 cells were determined by immunoblotting. All the expressions were normalized to β-actin. Data were exhibited as the mean ± SD (*n*=3). *P<0.05, **P<0.05, ***P<0.001 vs. LPS group; ^##^*P*<0.01, ^###^*P*<0.001 vs. Vehicle group.

Intestinal epithelial tight junction is a continuous ring-like structure that maintains the function of intestinal epithelial barrier. Besides, it has many functions, such as maintaining normal cell polarity, connecting two different intestinal epithelial cells, filling the gap between cells, regulating substance transportation, etc [35]. As a cell line established from colon adenocarcinoma, Caco-2 cell line is often used to study the intestinal barrier function *in vitro* since its polarity, tight junction structure, and energy absorption function are very similar to those of intestinal epithelial cells [36]. In addition, as important indicators of intestinal barrier function, JAM-A, ZO-1, Occludin and Claudin-1 proteins are often used as marker proteins of intestinal barrier function [37]. As shown in Figure 12A, pinocembrin promoted the mRNA expressions of Occludin, Claudin-1 and JAM-A in a dose-dependent manner in Caco-2 cells. Furthermore, the protein expressions of ZO-1, Occludin and Claudin-1 were decreased after LPS stimulation (Figure 12B,C). However, pinocembrin promoted the expressions of these proteins remarkably in a dose-dependent manner, indicating its improvement on the tight junction structure and promotion on the intestinal mucosal barrier function.

## Discussion

Since the etiology and pathogenesis of UC remain unknown, WHO has listed UC as one of the modern refractory diseases [38]. Oral administration of DSS to mice can induce severe colitis characterized by progressive weight loss, bloody diarrhea, large ulcer formation, diffuse necrosis, intense transmural inflammation and neutrophils infiltration, which symptoms are similar to human UC [39]. Our results showed that pinocembrin markedly improved the symptoms of DSS-induced colitis in mice. Moreover, no toxic reaction was observed in pinocembrin alone treatment mice, which suggests the relative safety and potential translational application for pinocembrin.

In recent years, increasing studies have indicated that dysbacteriosis is involved in the pathogenesis of UC. Previous studies indicated that the intestinal flora of UC patients is seriously dysregulated, which is related to a reduced abundance of probiotic bacteria *Firmicutes* and *Bacteroidetes*, and an increased abundance of pathogenic bacteria *Proteobacteria* [40,41]*.* In our research, the 16S rRNA sequencing was performed to detect the fecal bacteria and to assess whether the effect of pinocembrin in alleviating UC is linked to an improvement of bacterial community composition. Ultimately, we found that the predominant intestinal bacteria profiles were greatly shifted in DSS model group showing a significant decrease in fecal microbial flora diversity, which included a decrease in the commensal intestinal bacteria (such as *Firmicutes* and *Bacteroidetes*) and an increase in maleficent bacteria (such as *Proteobacteria*). In contrast, pinocembrin treatment significantly mitigated these parameters induced by DSS treatment.

Furthermore, it has been revealed that dysbacteriosis relates intimately with intestinal mucosal barrier damage [42]. Previous studies showed that intestinal integrity is injured in UC patients and DSS colitis mice, and gut barrier dysfunction leads to bacterial invasion and excessive inflammation, thereby further destroys barrier integrity [42,43,44]. In brief, the damage of intestinal mucosal barrier is an essential part of triggering above inflammatory feedback cycle. Our results suggest that pinocembrin may alleviate DSS colitis by improving the bacterial community composition. Moreover, we chose Caco-2 cells, a cell line with features similar to human intestinal epithelial cells [36], to explore the effects of pinocembrin on the expression of tight junction proteins (ZO-1, Occludin, Claudin-1) in intestinal mucosal barrier *in vitro*. Interestingly, pinocembrin apparently increased the expression levels of tight junction proteins and significantly reversed LPS-induced low expression of tight junction proteins in Caco-2 cells.

TLRs receptors are existed in intestinal epithelial cells and multiple immune cells, such as macrophages and dendritic cells, and play crucial roles in the intestinal defense against the invasion of pathogenic microorganisms [42]. TLR4, as one of the earliest discovered and most deeply studied of these receptors, association with its co-receptor myeloid differentiation factor MD2, is responsible for the physiologic recognition of many exogenous ligands (e.g. bacterial flagellin, LPS, etc.) [27]. TLR4 activation leads to the activation of NF-κB through the myeloid differentiation factor MyD88-dependent and/or Myd88-independent pathway and subsequently produce pro-inflammatory cytokines (e.g. COX-2, iNOS, TNF-α, etc.) [45]. Besides, there is a tremendous amount of research shows that the high expression of inflammatory mediators and cytokines plays essential character in the onset of UC [32]. In our data, pinocembrin remarkably suppressed the mRNA expression of TLR4, Myd88, iNOS, COX-2 and TNF-α, as well as the increased protein expression of TLR4 and phosphorylated NF-κB p65 in the colon of DSS colitis mice. Furthermore, we found that pinocembrin inhibited LPS-stimulated NF-κB p-p65 nuclear translocation and NF-κB-luciferase transcription. The anti-inflammatory effects of pinocembrin might be related to the blockade of the formation of LPS-induced TLR4/MD2 complex through binding to MD2 pocket, which ultimately suppressed the expression and release of cytokines and inflammatory mediators. These results demonstrated that inhibiting the excessive activation of TLR4/MD2/NF-κB signaling pathway contributed to the anti-inflammatory effects of pinocembrin in DSS-induced colitis.

Indeed, host health depends on a fine intestinal homeostasis between the innate/adaptive immune system and the microbiome. A number of studies suggest that a cross-talk interaction between gut microbiota and mucosal immune system contributes to the pathogenesis of IBD. On the one hand, the bacterial composition determines the region-specific expression of TLR4 [46,47], and the shift in the makeup of the intestinal microbiota induces a dysregulation of the intestinal immune response during IBD disease process [48]. On the other hand, both low and excessive TLR4 signaling influences the microbial composition, which could exacerbate the intestinal inflammation [49]. Hence, gut microbiota composition and TLR4 signal can serve as a mutual cause and consequence in the IBD pathogenesis. There is a need for research to precisely describe the regulation regarding cause and effect concerning gut microbiome and TLR4 signal in IBD pathogenesis. Additionally, the double-edged sword of TLR4 function in the intestinal immune homeostasis [47,50] makes the role of TLR4 in IBD pathogenesis intriguing and needs further clarification.

In conclusion, these new findings indicated that pinocembrin alleviated DSS-induced colitis in mice and administration of pinocembrin could ameliorate the intestinal damage, diarrhea and bloody stool. The anti-colitis mechanism of pinocembrin might be associated with improving the disturbed gut microbiota composition, suppressing TLR4/MD2/NF-κB signaling cascades, and preserving intestinal barrier integrity. These results suggest that pinocembrin could be a potential pharmaceutical candidate to ameliorate UC.

## Abbreviations

CD: Crohn's disease
DAMP: damage-associated molecular pattern
DSS: dextran sulfate sodium
H&E: hematoxylin and eosin
IBD: inflammatory bowel disease
iNOS: induced nitric oxide synthase
IκBα: inhibitor of nuclear factor–kappa B
JAM-A: junctional adhesion molecules
LPS: lipopolysaccharides
MD2: myeloid differentiation-2
MyD88: myeloid differentiation factor88
NF-κB: nuclear factor-kappa B
NO: nitric oxide
OD: optical density
OTU: operational taxonomic unit
PAMP: pathogen-associated molecular pattern
PCA: principal components analysis
PCR: polymerase chain reaction
RT: reverse transcriptase
SDS: sodium dodecyl sulfate
TLR4: Toll-like receptor-4
UC: ulcerative colitis
ZO-1: Zonula-occludens-1
